# Supplementary material for: Developing deep learning-based strategies to predict the risk of hepatocellular carcinoma among patients with nonalcoholic fatty liver disease from electronic health records
Source: medRxiv. 2023 Nov 17:2023.11.17.23298691. Preprint. [Version 1] doi: 10.1101/2023.11.17.23298691 (PMC10680899; doi:10.1101/2023.11.17.23298691)
Supplement: Supplement 1 [file media-1.pdf]

Supplementary Materials

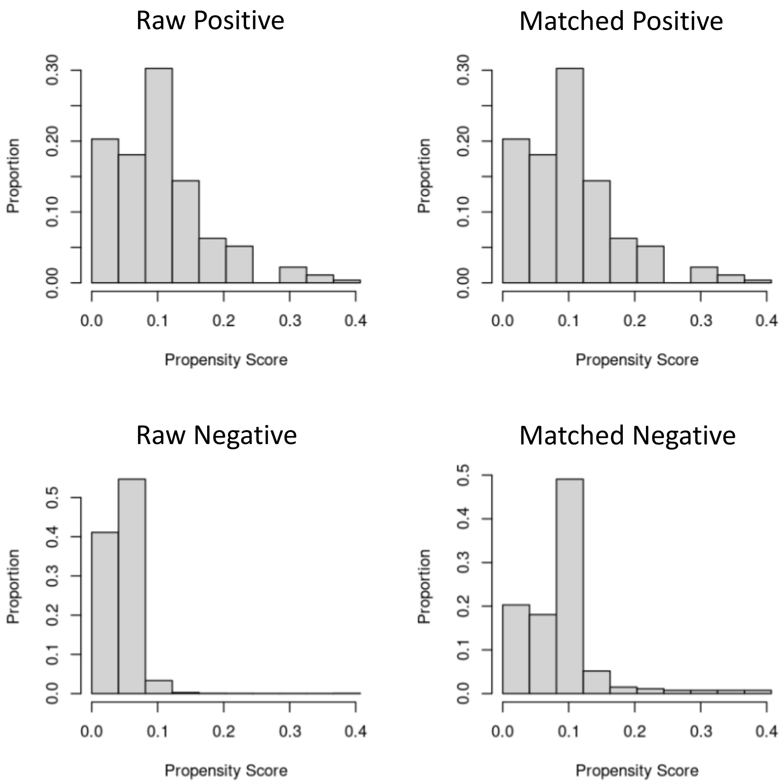

Figure S1. The distribution of propensity scores for positive and negative samples before and after propensity score matching. The distribution of the matched negative samples is closer to the positive samples.

RETAIN model

A

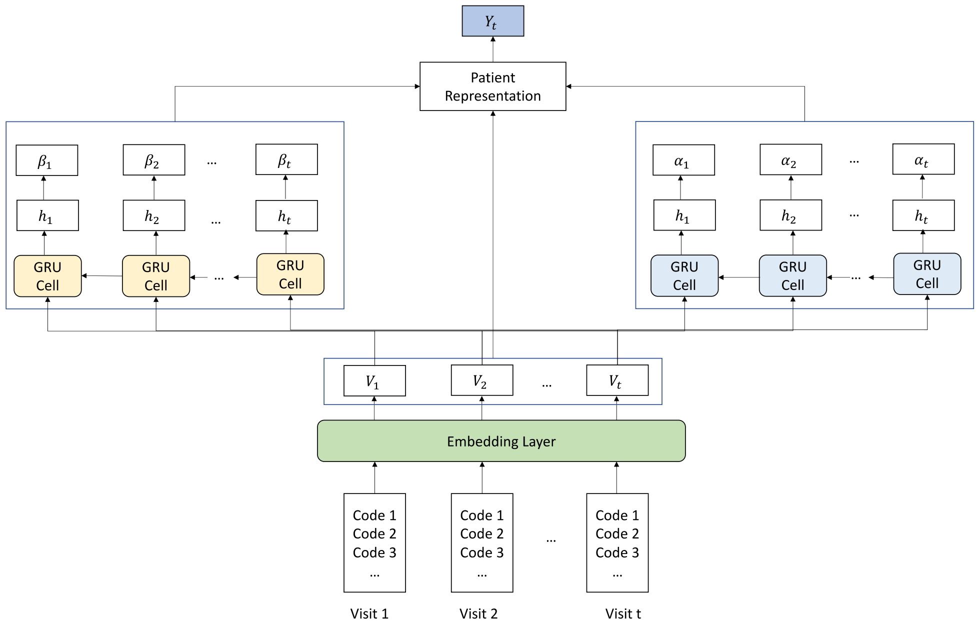

B

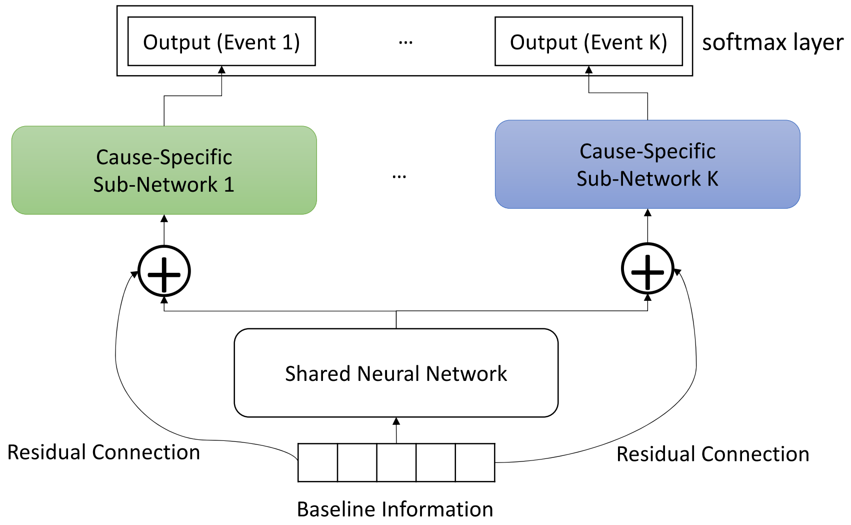

Figure S2. (A) The overview of RETAIN model. (B) The architecture of DeepHit model.

A common approach for analyzing time-to-event data with a classifier is to use the event status alone while ignoring the event times. Instead, we derive class labels by examining the event status at a chosen time point while removing patients who have insufficient follow-up (**Figure 2**). Specifically, the classification problem in this study is to predict NAFLD patients who will develop HCC within 10 years and those who will not, and the main objective is to identify risk factors and protective factors associated with the occurrence of HCC. We showed with Monte Carlo simulations that analyzing time-to-event data as a classification problem in this way allows us to identify risk factors reliably with strong control of type I error (Supplementary **Figure S1-2**). Further, under many conditions, the frequency of the positive class label is a well-calibrated estimator of the disease risk at the chosen time point (Supplementary **Figure S3**), although we do not need this property to identify risk factors. Accordingly, with this formulation, we can compare the performances of DeepHit and RETAIN in terms of predicting whether patients in a defined cohort develop a disease of interest within a specified time.

The formulation of disease prediction as a classification problem enabled us to take advantage of powerful deep learning models not developed for time-to-event data with censoring. We reframed the time-to-event problem as a classification problem by assigning class labels based on whether the event has occurred within a pre-specified time threshold, using the observed event times and statuses. We showed this formulation has desirable statistical properties in terms of the high accuracy of identifying risk factors and the calibration of the class probability as an estimator for the disease risk. This formulation thus allowed us to apply the RETAIN deep learning model in addition to the more statistically rigorous DeepHit model to identify risk factors for HCC progression among NAFLD patients, as well as exploring sex-specific patterns

of HCC progression. It also makes it convenient to incorporate longitudinal information in the classification algorithm without specialized models for longitudinal data.

Our classification framework does not consider competing risks, so the identified risk factors must be interpreted carefully. For example, we showed that being a non-smoker appeared to be a risk factor for HCC in a NAFLD patient, which is inconsistent with a prior large retrospective study. A more reasonable interpretation would be that being a non-smoker lowers the competing risk of death and thus allows NAFLD patients more time to develop HCC. Moreover, to estimate disease risk accurately in this setting, we continue to advocate the use of competing risk models for now, especially those that can account for changes in covariate values over time. The situation could be handled by considering a new, multi-category classification algorithm in the future. Nonetheless, our formulation of disease prediction as a classification problem facilitates the application of powerful longitudinal deep learning models that do not model event censoring and thus provides timely clinical insights into disease progression over time. We expect that this algorithm works best in common situations where there are notable changes in a patient's health in the years close to the clinical event.

We showed that powerful predictive models such as deep learning can be sensitive to covariate imbalance, such as sex bias. The performance of disease prediction decreased by > 5% when RETAIN was trained data from on one sex and tested on data for the other sex. This performance reduction occurs because RETAIN can identify sex-specific features. For example, in female patients with NAFLD, analysis of the attention weights in the trained RETAIN models revealed that rheumatoid arthritis may be a risk factor and kidney stones may be a protective factor for HCC progression. Our results thus indicate that using a sex-biased dataset for training can reduce the predictive performance and generalizability of the trained deep learning model to

other datasets. This finding can also be applied to other disease prediction tasks where sex and other patient characteristics such as race and ethnicity play important roles in disease progression.

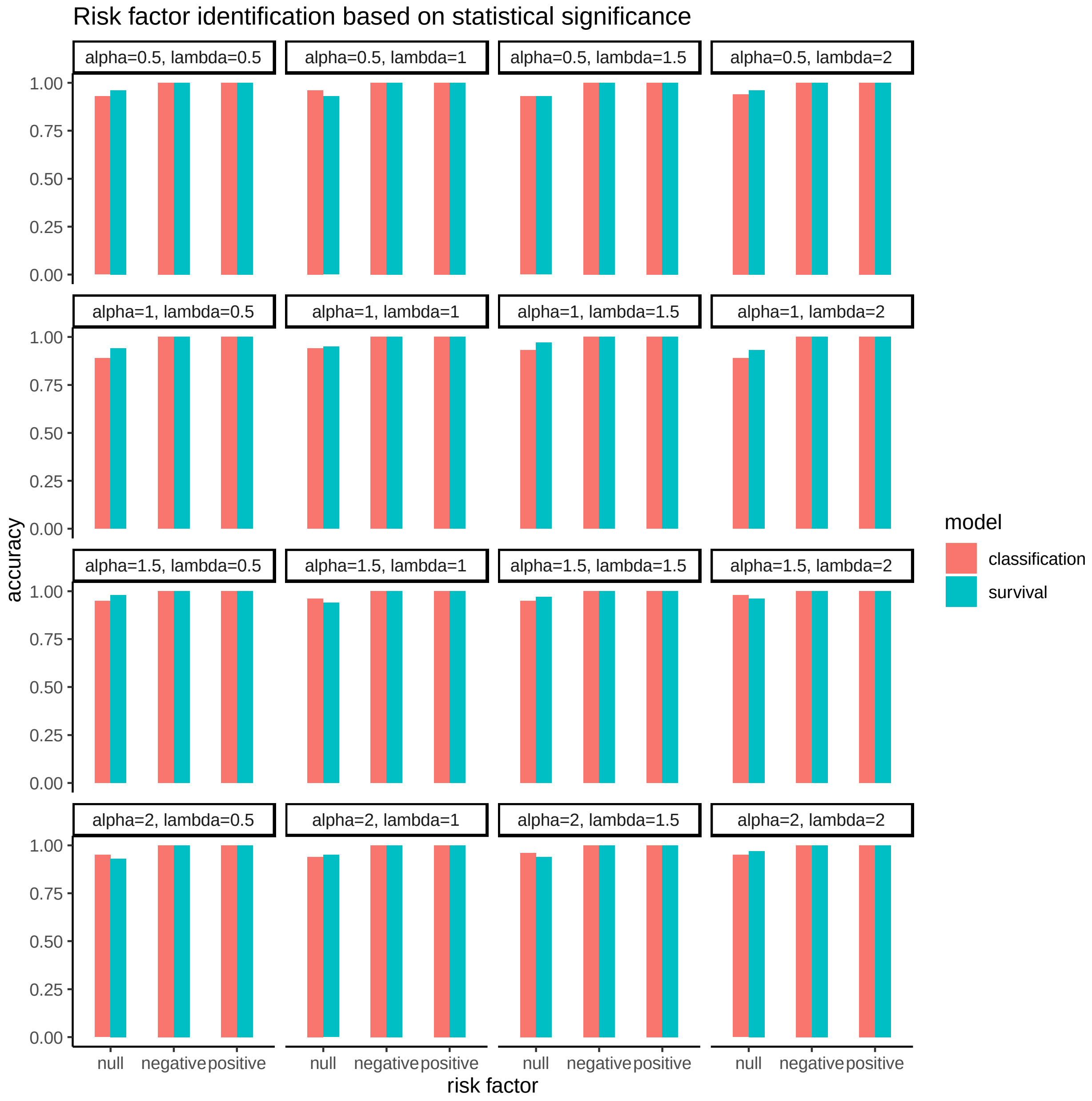

Figure S3. Comparison of the accuracy of risk factor identification under a survival model vs. a classification model. Each bar represents the accuracy of identifying a null, negative, or positive risk factor over 100 rounds of simulation based on whether the indicated model identified the

risk factor as statistically significant. Time-to-event data (n = 1000) were generated with event times following the Weibull distribution with various shape parameters (alpha) and rate parameters (lambda), under a uniform censoring scheme. The hazard rates were modified multiplicatively by a null, negative, or positive risk factor. The time-to-event analysis used the Cox proportional-hazards regression to identify risk factors, while the classification analysis used the logistic regression for which class labels were defined as event occurrence within a time cutoff. Results are shown for the cutoff set at the 75% quantile of observed event times, and similar results were obtained for cutoff set at the 25% and 50% quantiles.

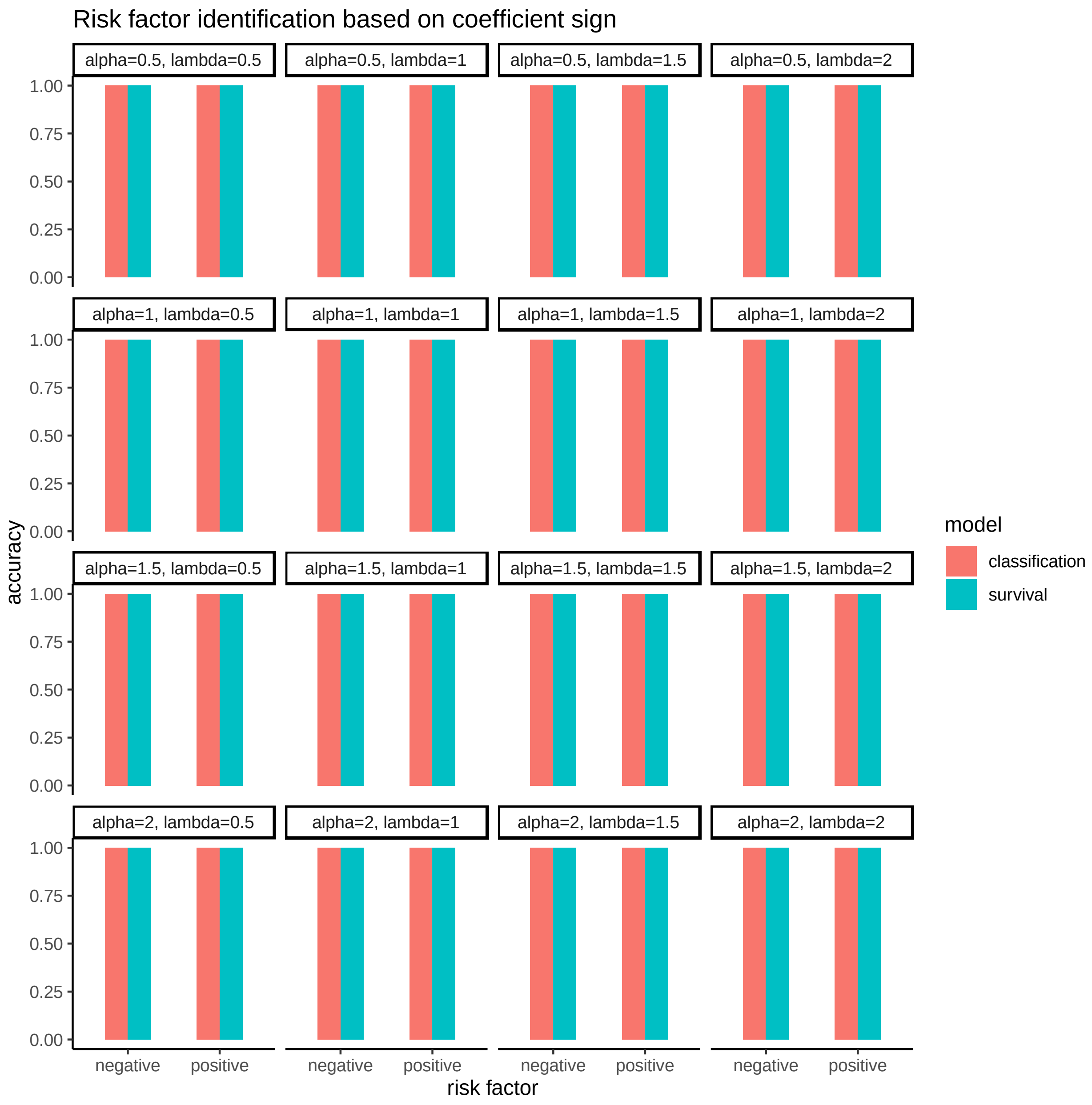

Figure S4. Comparison of the accuracy of risk factor identification under a survival model vs. a classification model, using the signs of the risk factor coefficient estimates. Each bar represents the accuracy of identifying a negative or positive risk factor over 100 rounds of simulation based on whether the indicated model estimated the coefficient for the risk factor with the correct sign (in the correct direction).

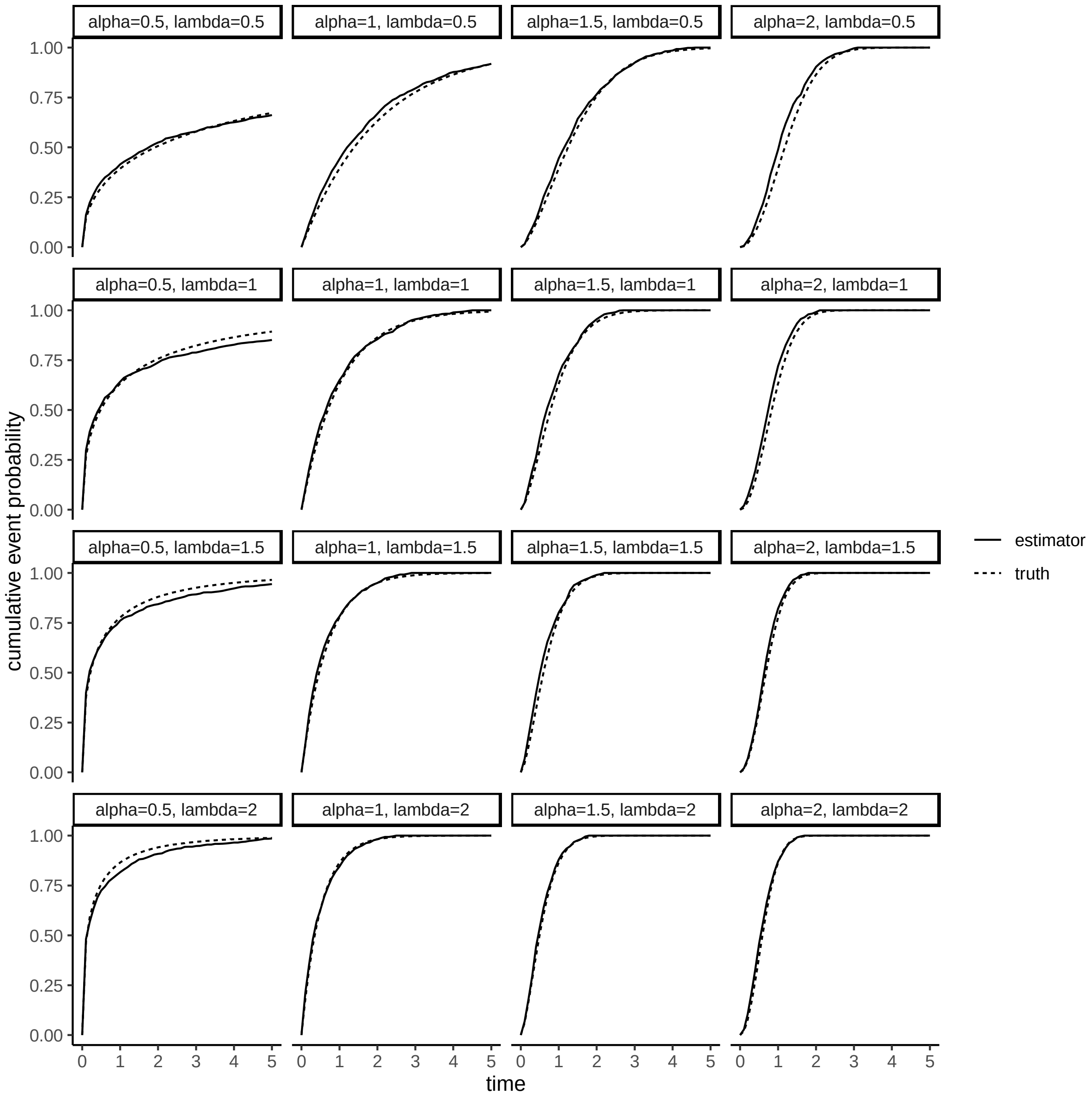

Figure S5. Class label probability is a well-calibrated estimator of the cumulative event probability under various conditions. Time-to-event data were generated using different Weibull distributions under a uniform censoring scheme. Class labels were defined based on whether the event was observed to occur before each time point.

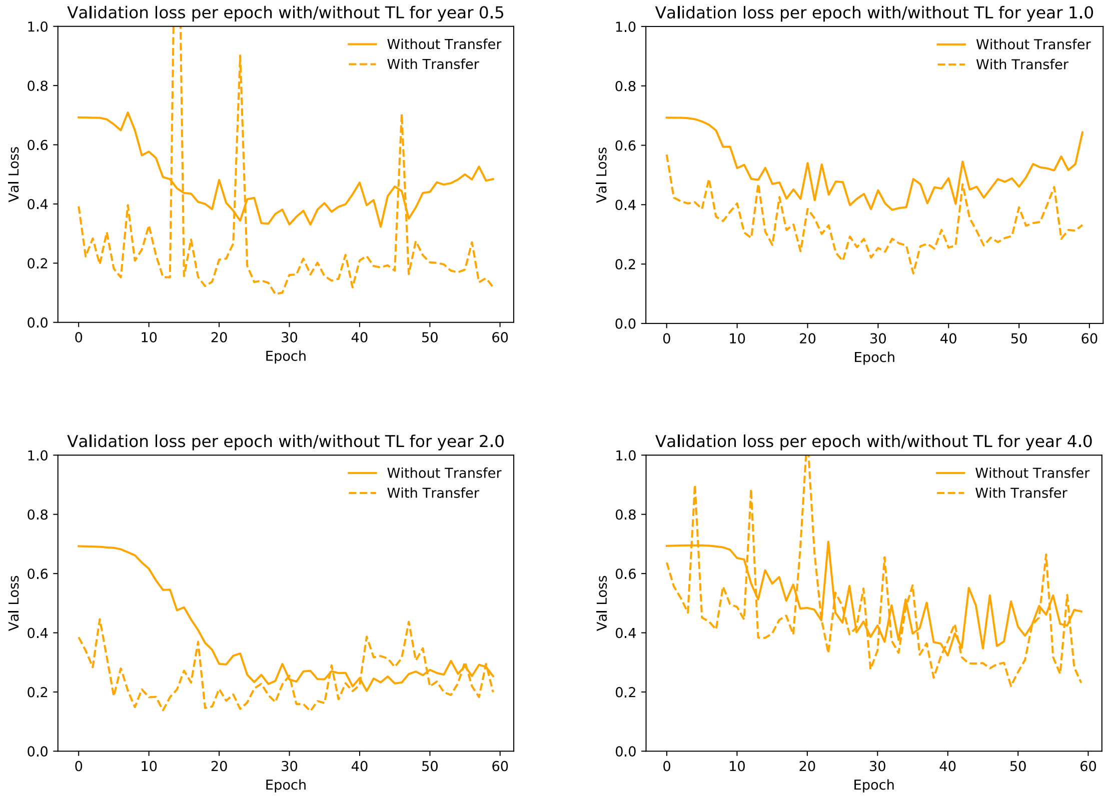

Figure S6. The validation loss with and without transfer learning under different masking length.

Table S1. Baseline variables for DeepHit.

Name	Type
Marital status	multiple
Race	multiple
tobacco_use_index	binary
diabetes_index	binary
hypertension_index	binary
thrombocytopenia_index	binary
athero_index	binary
hypothyroidism_index	binary
CKD_index	binary
COP_index	binary
hyperlipidemia_index	binary
cirrhosis	binary
high_fib4	binary
p_high_fib4	binary
obesity	binary

Table S2. High contribution medical codes with positive attention for male patients.

rank	code	annotation
0	SMOKE_1	Use tobacco
1	BMI_E	35.0<bmi<40.0
2	DIAG_Z51.89	Encounter for other specified aftercare
3	MED_ONDANSETRON	
4	MED_PROPOFOL	
5	LAB_1920-8_High	Aspartate aminotransferase
6	MED_SODIUM CHLORIDE	
7	SMOKE_0	No tobacco
8	MED_LVP SOLUTION	
9	DIAG_Z01.818	Encounter for other preprocedural examination
10	MED_MORPHINE	
11	DIAG_I10	Essential (primary) hypertension
12	DIAG_Z79.891	Long term (current) use of opiate analgesic
13	DIAG_E11.9	Type 2 diabetes mellitus without complications
14	MED_METOPROLOL	
15	BMI_ERROR	Null or negative
16	MED_ACETAMINOPHEN-OXYCODONE	
17	DIAG_F17.200	Nicotine dependence unspecified uncomplicated
18	MED_DOCUSATE-SENNA	
19	DIAG_Z95.1	Presence of aortocoronary bypass graft

Table S3. High contribution medical codes with positive attention for female patients.

rank	code	annotation
0	DIAG_M06.9	Rheumatoid arthritis unspecified
1	DIAG_K76.89	Other specified diseases of liver
2	DIAG_Z01.818	Encounter for other preprocedural examination
3	BMI_E	35.0<bmi<40.0
4	SMOKE_0	No tobacco
5	DIAG_E11.65	Type 2 diabetes mellitus with hyperglycemia
6	LAB_1920-8_Low	Aspartate aminotransferase
7	SMOKE_1	Use tobacco
8	DIAG_Z51.89	Encounter for other specified aftercare

9	DIAG_R19.7	Diarrhea unspecified
10	GEND_female	
11	MED_ONDANSETRON	
12	MED_SODIUM CHLORIDE	
13	DIAG_I10	Essential (primary) hypertension
14	LAB_1920-8_High	Aspartate aminotransferase
15	DIAG_R42	Dizziness and giddiness
16	DIAG_R05	Cough
17	DIAG_C78.7	Secondary malignant neoplasm of liver and intr...
18	BMI_C	25.0<bmi<30.0
19	MED_INSULIN GLARGINE	

Table S4. High contribution medical codes with negative attention for male patients.

rank	code	annotation
0	LAB_1920-8_In Control	Aspartate aminotransferase
1	MED_ZOLPIDEM	
2	MED_KETOROLAC	
3	A_J	Age_50
4	MED_DIPHENHYDRAMINE	
5	MED_ATORVASTATIN	
6	A_H	Age_40
7	MED_EPTIFIBATIDE	
8	A_I	Age_45
9	MED_MAGNESIUM HYDROXIDE	
10	MED_HYDROMORPHONE	
11	LAB_777-3_Within Range	Platelets
12	MED_ACETAMINOPHEN	
13	MARI_null	
14	MED_PANTOPRAZOLE	
15	MED_DOCUSATE	
16	DIAG_I20.0	Unstable angina
17	MED_STERILE WATER	
18	MED_AMLODIPINE	
19	MED_METOCLOPRAMIDE	

Table S5. High contribution medical codes with negative attention for female patients.

rank	code	annotation
0	DIAG_N20.0	Calculus of kidney
1	A_I	Age_45
2	MED_CIPROFLOXACIN	
3	A_J	Age_50
4	MED_PANTOPRAZOLE	
5	MED_KETOROLAC	
6	MED_AMLODIPINE	
7	MED_HYDROMORPHONE	
8	MED_STERILE WATER	
9	MED_LEVOFLOXACIN	
10	MED_DIPHENHYDRAMINE	
11	MED_CLINDAMYCIN	
12	A_K	Age_55
13	LAB_777-3_Within Range	Platelets
14	MED_DOCUSATE	
15	MED_ATORVASTATIN	
16	MED_METRONIDAZOLE	
17	MARI_null	
18	MED_ACETAMINOPHEN	
19	LAB_777-3_Low	Platelets

Inclusion and Exclusion Criteria for Non-alcoholic fatty liver disease (NAFLD) cohort from EHR

Inclusion:

1) patients were classified as having NAFLD if they had 2 or more elevated alanine aminotransferase (ALT) values (≥40 IU/mL for men and ≥31 IU/mL for women) in the ambulatory settings and more than 6 months apart, with no positive serologic testing for HBV (ie, HBV surface antigen) or HCV (ie, HCV RNA);

2) ≥18 years old at index date of follow-up (the date of first elevated ALT as the index date of follow-up for NAFLD cases).

Exclusion:

1) alcohol-related ICD-9 codes any time before or during study follow-up, ICD9: 571.0 Alcoholic fatty liver; 571.1 Acute alcoholic hepatitis; 571.2 Alcoholic cirrhosis of liver;571.3 Alcoholic liver damage, unspecified. ICD10: K70.0 Alcoholic fatty liver; K70.10 Alcoholic hepatitis without ascites; K70.30 Alcoholic cirrhosis of liver without ascites; K70.9 Alcoholic liver disease, unspecified;

2) positive AUDIT-C scores (≥4 in men and ≥3 in women) any time before or during study follow-up;

3) evidence of rare chronic hepatitides (eg, hereditary hemochromatosis, primary biliary cirrhosis, primary sclersoing cholangitis, a-1 antitrypsin disease, or autoimmune hepatitis) based on ICD-9 codes, ICD9: 713.0 Arthropathy associated with other endocrine and metabolic disorders (Code first underlying disease, as hemochromatosis [275.0]); 571.6 Biliary cirrhosis; 576.1 Cholangitis; 273.4 Alpha-1-antitrypsin deficiency; 571.42 Autoimmune hepatitis. ICD10: M14.80 Arthropathies in other specified diseases classified elsewhere, unspecified site; K74.3 Primary biliary cirrhosis; K83.0 Cholangitis; E88.01 Alpha-1-antitrypsin deficiency; K75.4 Autoimmune hepatitis.

Control cohort

Inclusion:

1) an ALT test performed;

2) no any documented liver-related risk factor: no NAFLD (persistently normal ALT); absence of positive tests for HBV and HCV; absence of alcohol ICD codes, ICD9: 571.0 Alcoholic fatty liver; 571.1 Acute alcoholic hepatitis; 571.2 Alcoholic cirrhosis of liver;571.3 Alcoholic liver

damage, unspecified, ICD10: K70.0 Alcoholic fatty liver; K70.10 Alcoholic hepatitis without ascites; K70.30 Alcoholic cirrhosis of liver without ascites; K70.9 Alcoholic liver disease, unspecified;

3) all AUDIT-C scores (<4 in men and <3 in women);

4) the date of first ALT test in the study timeframe as the index date of follow-up for controls;

5) random sampling without replacement (case: control= 1:1): sex; age at first ALT (index date); duration from their first visit to the first ALT test date (These three conditions of case are best the same as the control. If it is not the same, we can take the nearest one.).

Inclusion and Exclusion Criteria for Hepatocellular cancer (HCC) cohort from EHR

Inclusion:

1) patients diagnosed with HCC (4 ICD codes for HCC: 155.0, C22.0, C22.8 and C22.9)

2) ≥18 years old at first visit date

Exclusion:

1) patients were classified as having non-alcoholic fatty liver disease (NAFLD) if they had 2 or more elevated alanine aminotransferase (ALT) values (≥40 IU/mL for men and ≥31 IU/mL for women) in the ambulatory settings and more than 6 months apart (loinc code for ALT test: 1742-6)

Control cohort

Inclusion:

1) patients diagnosed without liver cancer (14 ICD codes for liver cancer: 155, 155.0, 155.1, 155.2, 197.7, 209.72, C22.0, C22.1, C22.2, C22.7, C22.8, C22.9, C78.7 and C7B.02)

2) ≥18 years old at first visit date

3) random sampling without replacement (case: control= 1:10): same gender; same age at first visit date; same duration from the first visit to the last visit date (If the sample size of the exact matched controls is not enough, please use the nearest to match).

Exclusion:

1) patients were classified as having NAFLD if they had 2 or more elevated alanine aminotransferase (ALT) values (≥40 IU/mL for men and ≥31 IU/mL for women) in the ambulatory settings and more than 6 months apart (loinc code for ALT test: 1742-6)
